# Regulation of glucose metabolism by p62/SQSTM1 through HIF1α

**DOI:** 10.1242/jcs.178756

**Published:** 2016-02-15

**Authors:** Ke Chen, Jin Zeng, Haibing Xiao, Chunhua Huang, Junhui Hu, Weimin Yao, Gan Yu, Wei Xiao, Hua Xu, Zhangqun Ye

**Affiliations:** 1 Department of Urology, Tongji Hospital, Tongji Medical College, Huazhong University of Science and Technology, Wuhan 430030, People's Republic of China; 2 Institute of Urology, Tongji Hospital, Tongji Medical College, Huazhong University of Science and Technology, Wuhan 430030, People's Republic of China; 3 College of Basic Medicine Science, Hubei University of Chinese Medicine, Wuhan 430065, People's Republic of China

**Keywords:** p62, HIF1α, CUL2, Protein stability, Transcriptional activity

## Abstract

The signaling adaptor sequestosome 1 (SQSTM1)/p62 is frequently overexpressed in tumors and plays an important role in the regulation of tumorigenesis. Although great progress has been made, biological roles of p62 and relevant molecular mechanisms responsible for its pro-tumor activity remain largely unknown. Here, we show that p62 knockdown reduces cell growth and the expression of glycolytic genes in a manner that depends on HIF1α activity in renal cancer cells. Knockdown of p62 decreases HIF1α levels and transcriptional activity by regulating mTORC1 activity and NF-κB nuclear translocation. Furthermore, p62 interacts directly with the von Hippel-Lindau (VHL) E3 ligase complex to modulate the stability of HIF1α. Mechanistically, p62 binds to the VHL complex and competes with HIF1α. Expression of p62 inhibits the interaction of DCNL1 (also known as DCUN1D1) with CUL2 and attenuates the neddylation of CUL2, and thus downregulates the VHL E3 ligase complex activity. Functionally, HIF1α expression is required for p62-induced glucose uptake, lactate production and soft agar colony growth. Taken together, our findings demonstrate that p62 is a crucial positive regulator of HIF1α, which is a facilitating factor in p62-enhanced tumorigenesis.

## Abstract (precis)

**Highlighted Article:** p62 is a crucial positive regulator of HIF1α, which is a facilitating factor in p62-enhanced tumorigenesis.

## INTRODUCTION

Renal cell carcinoma (RCC) is a common urologic cancer that accounted for about 143,000 deaths in 2012 (Li et al., 2013a; Frew and Moch, 2014). It is the 16th most common cause of death from cancer worldwide (Frew and Moch, 2014). Approximately 70–80% of adult renal cell carcinomas are classified as clear cell renal cell carcinomas (ccRCCs) (Sato et al., 2013). Genetically, ccRCC is usually linked to loss of chromosome 3p [harbors von Hippel-Lindau (*VHL*), *PBRM1*, *BAP1* and *SETD2*] (Creighton et al., 2013; Li et al., 2013b). Mutations in the *VHL* tumor suppressor gene account for up to 70% of hereditary ccRCCs (Kaelin and Maher, 1998). In addition to loss of chromosome 3p, gains of chromosome 5q35.3 have been demonstrated in about 70% of ccRCCs (Beroukhim et al., 2009; Chen et al., 2009; Hagenkord et al., 2011; Shen et al., 2011; Dondeti et al., 2012; Li et al., 2013b). Furthermore, the p62 transcript has been mapped to this region (Li et al., 2013b). p62 is classically known as a scaffold protein that participates in regulation of many cellular processes, for example, cell proliferation and growth, malignant transformation, apoptosis, inflammation and autophagy (Mathew et al., 2009; Moscat and Diaz-Meco, 2009, 2011, 2012; Bitto et al., 2014). The best-known function of p62 is in the targeting polyubiquitylated proteins for autophagy-mediated degradation through interaction with ubiquitin and LC3 (Kirkin et al., 2009; Moscat and Diaz-Meco, 2009; Lin et al., 2013). In addition, p62 regulates NF-κB activation by interacting with atypical protein kinase C (aPKC) isoforms, MEKK3, RIP1 kinase and TRAF6 (Sanz et al., 1999, 2000; Wooten et al., 2005; Duran et al., 2008; Nakamura et al., 2010). p62 also has a central role in the mammalian target of rapamycin complex (mTORC1) pathway by binding with mTOR, raptor and TRAF6 (Duran et al., 2011; Linares et al., 2013). Additionally, p62 binds to the NRF2-binding domain of Keap1 and competes with NRF2 for binding, leading to upregulation of NRF2 and NRF2 target genes (Komatsu et al., 2010; Ryoo et al., 2015). Deregulation of NF-κB, mTORC1 and NRF2 signaling pathways are crucial factors that contribute to the initiation and/or progression of various malignancies. p62 expression is upregulated in multiple malignancies, including breast tumors, lung adenocarcinomas, lung squamous cell carcinomas, hepatocellular carcinomas and ccRCCs (Thompson et al., 2003; Moscat and Diaz-Meco, 2009; Inoue et al., 2012; Li et al., 2013b; Bao et al., 2014). Notably, p62 expression correlates with higher tumor grades in ccRCCs (Li et al., 2013b). However, Wei et al. report that deletion of FIP200 results in autophagy inhibition and p62 accumulation, leading to decreased mammary tumorigenesis (Wei et al., 2011). It has also been reported that p62 is downregulated in the stroma but overexpressed in the epithelial compartment of human primary prostate tumors (Valencia et al., 2014). The downregulation of p62 in stromal fibroblasts results in increased tumorigenesis of epithelial prostate cancer cells though the mTORC1–Myc–IL-6 pathway (Valencia et al., 2014). Thus, p62 can both promote and suppress tumorigenesis, depending on the tumor microenvironment.

Metabolic reprogramming is a hallmark of cancer cells. An increasing number of studies has revealed that p62 plays key roles in regulating energy metabolism. Downregulation of p62 leads to decrease in ATP and lactate levels by regulating mitochondrial F_1_ Fo-ATP synthase activity in glioblastoma stem cells (Galavotti et al., 2013). p62^−/−^ mice show a significantly reduced metabolic rate, indicated by decreased oxygen consumption (Rodriguez et al., 2006; Lee et al., 2010). In addition, adipocyte-specific p62^−/−^ mice also exhibit a significantly reduced metabolic rate caused by impaired mitochondrial function (Müller et al., 2013). Moreover, Valencia et al. have found that p62-knockout fibroblasts exhibit decreased glucose uptake and lactate secretion (Valencia et al., 2014). Because of its complexity, the function and the underlying mechanism of p62 in tumorigenesis and metabolic reprogramming remain to be investigated. Here, we address the role of p62 in glucose metabolism and in the growth of renal cancer cells in soft agar. Mechanistically, p62 induces hypoxia-inducible-factor 1α (HIF1α) signaling though the upregulation of mTORC1 and NF-κB activity, and the downregulation of VHL E3 ubiquitin ligase activity, thus inducing HIF1α activation, glucose uptake and lactate production, and growth in soft agar.

## RESULTS

### p62 knockdown reduces cell growth and the expression of glycolytic genes in renal cancer cells

**Fig. 1.**
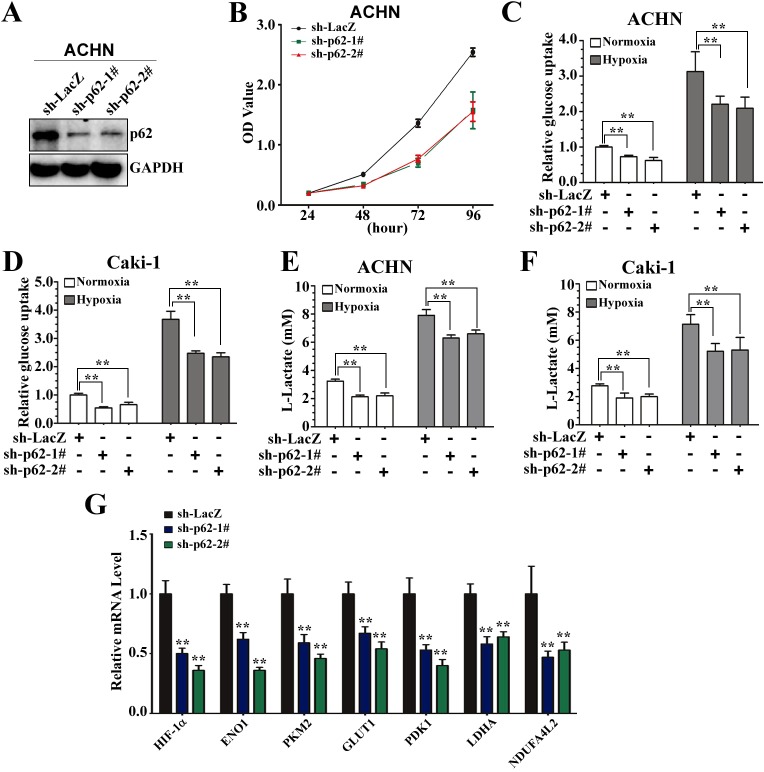
**p62 is crucial for cell proliferation and promotes glucose metabolism.** (A) Generation of p62-knockdown ACHN cells. ACHN cells were infected with lentiviruses expressing shRNA against p62 (sh-p62; two different oligonucleotides) or LacZ (sh-LacZ). Immunoblotting was performed to evaluate the expression of p62. GAPDH is an internal control. (B) A CCK-8 kit was utilized to quantify cell viability at indicated time points. Data presented are means±s.d. from three independent experiments. (C,D) Knockdown of p62 reduces glucose uptake under both normoxia and hypoxia in ACHN and Caki-1 cells. ACHN (C) or Caki-1 (D) cells stably expressing sh-p62-1#, sh-p62-2# or sh-LacZ were grown for 48 h. The cells were then subjected to normoxic or hypoxic conditions for 12 h, and glucose uptake was measured and expressed as a ratio of the levels in sh-LacZ cells. **Significant differences, *P*<0.01 (Student's *t*-test). (E,F) Cells were treated as in C, and extracellular lactate in the culture medium was measured and expressed as a ratio of the levels in sh-LacZ cells. **P<0.01. (G) Knockdown of p62 decreases the expression of genes involved in glucose metabolism. The relative expression of genes (HIF1α, ENO1, PKM2, GLUT1, PDK1, LDHA and NDUFA4L2) involved in glucose metabolism in ACHN cells expressing sh-p62-1#, sh-p62-2# or sh-LacZ was measured using qRT-PCR analysis. Expression was normalized to that in cells expressing sh-LacZ (control); data are plotted as the mean±s.d. of three independent experiments. **P<0.01 versus control (Student's *t*-test).

Previous studies have indicated that p62 is highly expressed in ccRCC lines and tumors (Li et al., 2013b). Here, we further investigated a role of p62 in the Warburg effect in ccRCC lines. We first constructed isogenic pairs of ACHN cells differing only in p62 expression levels using stable RNA knockdown (sh-p62 lines) (Fig. 1A). Consistent with previous reports that knockdown of p62 decreases the viability of ccRCC cell lines, the two newly established ACHN cells expressing sh-p62 also grew more slowly than the control ACHN cells (Fig. 1B). We therefore explored whether p62 regulates the glucose metabolism in ccRCC lines. We found that the p62-knockdown cells had significantly reduced glucose uptake (Fig. 1C,D) and lactate production (Fig. 1E,F) in both ACHN and Caki-1 cell backgrounds under both normoxia and hypoxia. The efficiency of small hairpin (sh)RNA-mediated knockdown of p62 in ACHN and Caki-1 cells was examined by western blotting (Fig. S1A). An increasing number of studies indicates that metabolic alterations are regulated by HIF1α and a series of glycolytic genes, for example, glucose transporter (*GLUT1*), enolase 1 (*ENO1*), *PKM2*, lactate dehydrogenase A (*LDHA*), pyruvate dehydrogenase lipoamide kinase isozyme 1 (*PDK1*) and *NDUFA4L2* (Semenza, 2010; Tello et al., 2011). We next examined the expression profiles of all these genes using real-time quantitative PCR (RT-qPCR). The mRNA expression levels of all these genes were decreased in p62-knockdown ACHN cells (Fig. 1G). Taken together, these results suggest that p62 knockdown inhibits glucose metabolism, most probably through regulation of the HIF pathway in renal cancer cells.

### p62 is a positive regulator of HIF1α

**Fig. 2.**
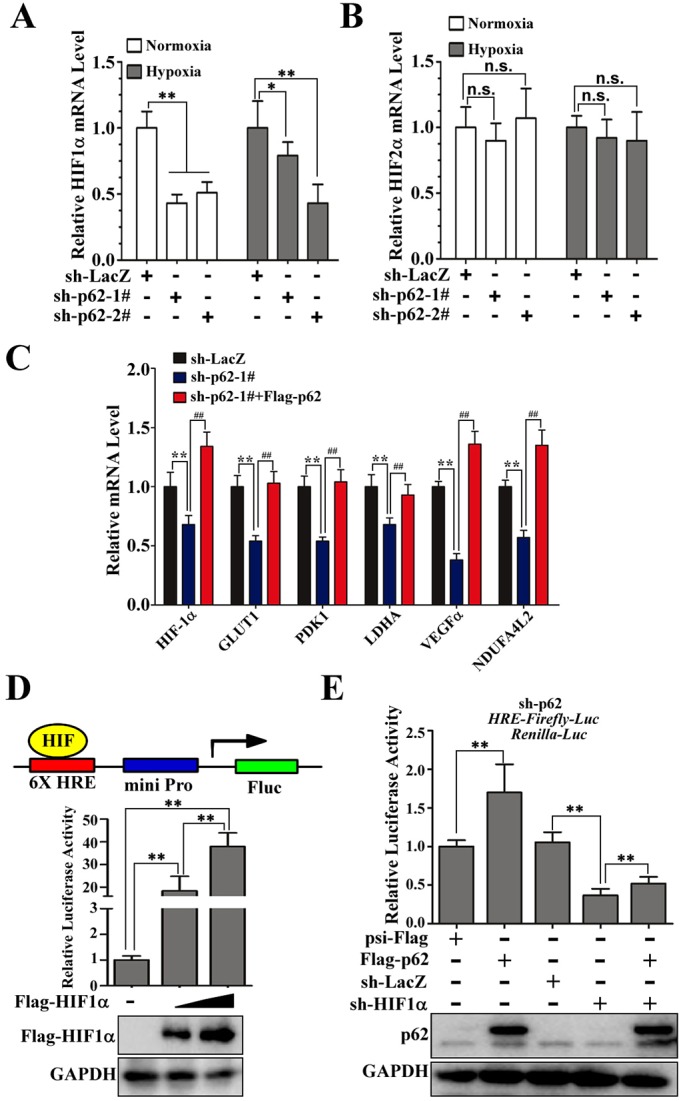
**p62 is a positive regulator of HIF1α.** (A,B) p62 knockdown reduces HIF1α mRNA without affecting HIF2α mRNA in cells both under normoxia and hypoxia. The relative expression of HIF1α and HIF2α in ACHN cells expressing sh-p62-1#, sh-p62-2# or sh-LacZ under normoxia and hypoxia was measured by using qRT-PCR analysis. Expression was normalized to that in cells expressing sh-LacZ (control); data are plotted as the mean±s.d. of three independent experiments. n.s., not significant (*P*>0.05); *P<0.05 versus control; **P<0.01 versus control (Student's *t*-test). (C) Relative expression of HIF-1α, GLUT1, PDK1, LDHA, VEGFα and NDUFA4L2 mRNA levels in control (LacZ), p62-knockdown or p62-rescued (sh-p62-1#+Flag-p62) ACHN cells, as determined by using RT-qPCR analysis. **P<0.01 versus control; ^##^*P*<0.01 versus sh-p62-1#. (D) HRE–Fluc construct and luciferase activity measurement. ACHN cells were infected with lentivirus expressing 6×HRE-Fluc and SV40-Rluc (internal control, for normalization) luciferase reporter genes to generate HRE-reporter cells stably expressing firefly luciferase, controlled by HIF1. HRE-reporter cells were transiently transfected with Flag–HIF1α (0, 1 or 2 μg). After transfection for 36 h, luciferase fluorescence was measured and normalized to that of the internal control. Western blotting analysis of the whole-cell lysates from ACHN cells was performed to evaluate the expression of Flag-HIF1a. GAPDH is an internal control. **P<0.01 versus control (Student's *t*-test); *n*=3. (E) p62 promotes 6×HRE-driven luciferase activity that is dependent on HIF1α in ACHN cells. HRE-reporter ACHN cells stably expressing sh-p62 were infected with different combinations of lentivirus encoding psi-Flag, Flag–p62, sh-LacZ and sh-HIF1α. After 72 h of culture, luciferase fluorescence was measured, and the ratio of Fluc to Rluc activity was normalized to that of cells expressing psi-Flag control plasmids (mean±s.d.; *n*=3). Western blotting analysis was performed to evaluate the expression of Flag-p62. GAPDH is an internal control. **P<0.01 versus control (Student's *t*-test).

Because HIF1 and HIF2 are central players in the transcriptional activation of genes that encode proteins involved in glucose and energy metabolism (Kim et al., 2006), we next analyzed whether p62 influenced HIF1α and HIF2α transcription. Here, p62 knockdown decreased the levels of HIF1α mRNA without affecting HIF2α mRNA under both normoxic and hypoxic conditions (Fig. 2A,B). Thus, we hypothesized that p62 is involved in glucose metabolism though the regulation of HIF1α activity and that increased HIF1α activity could, in turn, induce the transcription of several glycolytic genes. Indeed, the mRNA levels of HIF1α and several HIF1α target genes were all reduced specifically in p62-knockdown cells and restored by rescue of p62 expression under normoxic conditions (Fig. 2C). To test whether positive regulation of HIF1 by p62 affects HIF transcriptional activity, we established ACHN cells that stably expressed firefly luciferase under the hypoxia response element (6×HRE-FLuc) and a *Renilla* luciferase SV40 reporter (SV40-RLuc; internal control), and then transiently transfected these engineered cells with increasing concentration of Flag–HIF1α. As expected, expression of HIF1α promoted the transcriptional activation of the 6×HRE­Fluc reporter gene in a dose-dependent manner (Fig. 2D), indicating that the HRE-reporter cells are a suitable system. Next, in order to test the effects of p62 on the transcription of HRE-regulated genes, the HRE-reporter cells were infected with an shRNA against p62 (sh-p62-1#), and then these engineered cells were infected with different combinations of lentiviral vectors that encoded Flag–p62, an shRNA against LacZ (sh-LacZ) or an shRNA against HIF1α (sh-HIF1α). Luciferase activity analysis of the dual luciferase reporter revealed that overexpression of p62 resulted in increased HRE-mediated transcription, which was restored (or in fact significantly repressed) by concurrent expression of sh-HIF1α (Fig. 2E). Efficiency of sh-HIF1α-mediated knockdown was also verified using RT-qPCR analysis (Fig. S1B). Taken together, these results indicate that p62 can upregulate HIF1α levels, HIF transcriptional activity and expression of HIF1 target genes.

### p62-dependent HIF1α levels are regulated by mTORC1 and NF-κB

**Fig. 3.**
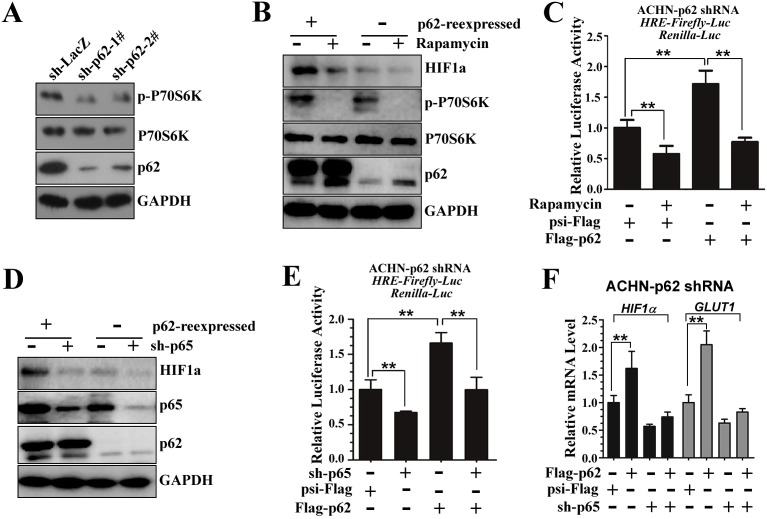
**p62-dependent HIF1α levels are regulated by mTORC1 and NF-κB.** (A) Stable p62 knockdown in ACHN cells downregulates the phosphorylation of P70S6K. Immunoblot for p62, p-P70S6K, p70S6K and GAPDH protein levels in whole-cell lysates from control (sh-LacZ) or p62-knockdown (sh-p62-1# or 2#) ACHN cells. (B) Rapamycin treatment reduces HIF1α protein levels in p62-rescued (re-expressed) ACHN cells. ACHN cells stably expressing sh-p62-1# were infected with lentivirus encoding psi-Flag or Flag–p62. After 72 h of culture, cells were treated with (+) or without (−) 20 nM of rapamycin for 24 h before cell lysis. Cell lysates were subjected to SDS­PAGE followed by immunoblotting with antibodies against p62, p-P70S6K, p70S6K and GAPDH. Because the HIF1α protein is unstable under normoxia, HIF1α was enriched through immunoprecipitation with an anti-HIF1α (rabbit polyclonal) antibody, and then the protein levels of HIF1α were determined by western blotting. (C) Rapamycin treatment downregulates p62-mediated enhancement of 6×HRE-driven luciferase activity. HRE-reporter ACHN cells were infected with lentivirus encoding sh-p62-1#. After 72 h of culture, the cells were transiently transfected with Flag–p62 and treated with or without rapamycin (20 nM) for 24 h, followed by measurement of the luciferase activity. **P<0.01 versus control (Student's *t*-test); mean±s.d.; *n*=3. (D) Knockdown of p65 reduces HIF1α protein levels in p62-rescued (re-expressed) ACHN cells. p62-knockdown cells were infected with different combinations of lentivirus that encoded Flag–p62 or/and sh-p65. After 72 h of culture, cell lysates were subjected to SDS­PAGE followed by immunoblotting with anti-­p62 and anti-­GAPDH antibodies. HIF1α was enriched through immunoprecipitation with anti-HIF1α (rabbit polyclonal) antibodies, and then the protein levels of HIF1α were determined by western blotting. (E) Knockdown of p65 downregulates p62-mediated enhancement of 6×HRE-driven luciferase activity. HRE-reporter ACHN cells were infected with lentivirus encoding sh-p62-1#. Cells were infected with different lentiviruses as in D. After 72 h of culture, the HRE luciferase activity was measured. **P<0.01 versus control (Student's *t*-test); mean±s.d.; *n*=3. (F) Inhibition of NF-κB signaling reduces p62-mediated upregulation of HIF1α and the mRNA levels of its target genes. The relative expression of HIF1α and GLUT1 in p62-knockdown stable ACHN cells that had been transfected with different combinations of Flag–p62, psi-Flag and/or sh-p65. Expression was normalized to that in cells expressing psi-Flag (control). **P<0.01 versus control Student's *t*-test); mean±s.d.; *n*=3.

Previous studies have indicated that p62 promotes mTORC1 activity to regulate nutrient sensing (Duran et al., 2011), and mTOR signaling has a role in regulating HIF1α protein expression (Thomas et al., 2006). As expected, stable p62 knockdown (sh-p62) downregulated the phosphorylation of p70S6K (p-P70S6K, an indicator of mTORC1 activity) (Fig. 3A). Western blot analysis showed that re-introduction of p62 into the knockdown cells upregulated HIF1α protein levels (Fig. 3B, lane 1 and 3), whereas the increase in HIF1α expression promoted by p62 was remarkably attenuated when cells were treated with the mTORC1 inhibitor rapamycin (Fig. 3B). Moreover, analysis of HRE-regulated transcription in the presence and absence of rapamycin indicated that the positive effect of p62 on HIF1α-induced transcription was remarkably dampened by rapamycin treatment (Fig. 3C), suggesting that the effect of p62 on HIF1α levels is mediated, at least in part, through mTORC1.

HIF1α has also been shown to be upregulated upon binding of NF-κB to the HRE, and p62 has been suggested to promote NF-κB activity; therefore, we next examined whether the NF-κB pathway is involved in p62-dependent regulation of HIF1α. Indeed, immunofluorescence and western blot analysis confirmed that p62 knockdown severely impaired nuclear translocation of NF-κB in ACHN cells (Fig. S2A,B). Depletion of p65 (also known as RELA) strongly impaired the p62-induced upregulation of HIF1α in normoxic ACHN cells (Fig. 3D). In addition, p62-induced upregulation of the transcriptional activities of HIF1α, measured by HRE-luciferase reporter activity assay, was also significantly reduced upon knockdown of p65 (Fig. 3E). Efficiency of sh-p65-mediated knockdown was verified by western blotting and RT-qPCR analysis (Fig. 3D; Fig. S2C). Consistently, the addition of the NF-κB inhibitor pyrrolidine dithiocarbamate (PDTC) also reduced HIF1α protein levels and HIF transcriptional activities in p62-rescued ACHN cells (Fig. S2D,E). Furthermore, the HIF1α and GLUT1 mRNA levels were both specifically elevated in p62-rescued ACHN cells, whereas these increases were severely impaired by the suppression of p65 (Fig. 3F). Collectively, these results demonstrate that both mTORC1 and NF-κB signaling contribute to p62-regulated increases in the levels of HIF1α.

### p62 interacts with the VHL E3 ubiquitin ligase complex

**Fig. 4.**
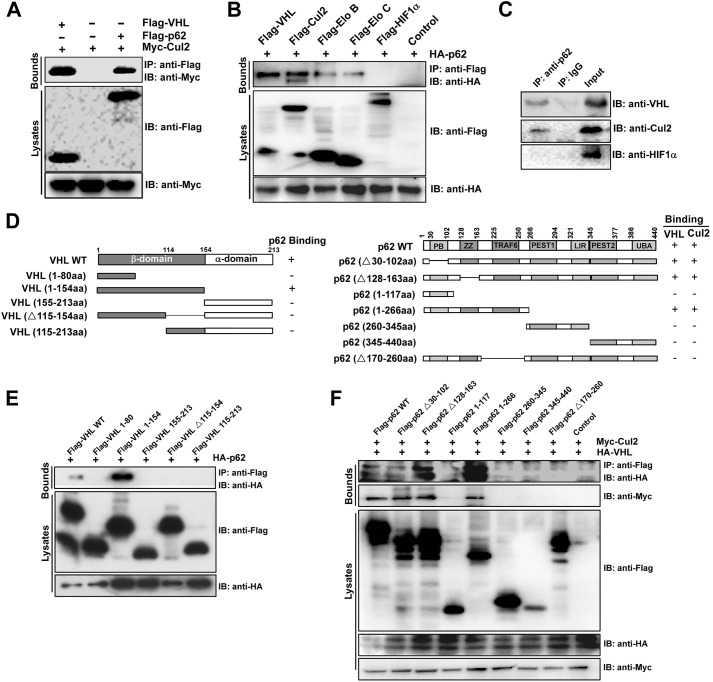
**p62 interacts with the VHL E3 ubiquitin ligase complex.** (A) p62 interacts with CUL2. Co-transfection of Myc–CUL2 into HEK293T cells was performed, together with Flag–VHL or Flag–p62. At 48 h after transfection, the whole-cell lysates were extracted for co-immunoprecipitation with an anti-Flag antibody, followed by probing for Myc. IB, immunoblotting; IP, immunoprecipitation. (B) HEK293T cells were co-transfected with HA–p62 together with Flag–VHL, Flag–CUL2, Flag–elonginB, Flag–elonginC, Flag–HIF1α or psi-Flag. At 48 h after transfection, the whole-cell lysates were extracted for co-immunoprecipitation with an antibody against Flag, followed by probing with an anti-HA antibody. (C) p62 interacts with VHL and CUL2 *in vivo*. The HEK293T cell lysates were immunoprecipitated (IP) with a control antibody (rabbit IgG) or an anti-­p62 antibody, and analyzed by immunoblotting (IB) with antibodies against VHL, CUL2 or HIF1α. (D) Domain structure and deletion constructs of VHL (left) and p62 (right). Numbers refer to amino acid residues. (E) Mapping of the p62 ­binding region of VHL. HEK293T cells were transiently transfected with HA–p62 along with various Flag-­tagged VHL deletion mutants, as indicated. The cell lysates were immunoprecipitated with an anti-­Flag antibody and immunoblotted with an anti-­HA antibody. (F) Mapping of the VHL- and CUL2-binding region of p62. HEK293T cells were transiently transfected with HA–VHL and Myc–CUL2 along with various Flag-tagged p62 deletion mutants, as indicated. The cell lysates were immunoprecipitated with anti-­Flag antibody and immunoblotted with anti-­HA and anti-Myc antibodies.

A previous study has indicated that p62 can co-immunoprecipitate with several proteins, including CUL2 (Tanji et al., 2012). Thus, we asked if p62-mediated upregulation of HIF1α protein levels is, in part, regulated through formation of a complex with the VHL E3 ligase, altering the HIF1α degradation pathway. To confirm the interaction of p62 with CUL2, we performed a co-immunoprecipitation assay, which showed that Myc–CUL2 co-precipitated efficiently with Flag–p62 in ACHN cells (Fig. 4A, lane 3). Furthermore, co-immunoprecipitation assays of VHL, CUL2, elongin B, elongin C and HIF1α with hemagglutinin (HA)-tagged p62 in ACHN cells showed that HA–p62 co-precipitated efficiently with VHL, CUL2, elongin B and elongin C but not with HIF1α (Fig. 4B). Bimolecular fluorescence complementation (BiFC) analysis has been widely used for visualization and identification of protein interactions in cells (Shyu et al., 2006, 2008). To verify that p62 interacts with VHL by using the BiFC system, p62 and VHL were fused to the N-terminal fragment of Venus truncated at residue 173 (VN173) and the C-terminal fragment of Venus truncated at residue 155 (VC155). No fluorescence was detected in cells that expressed either p62–VN173 or VHL–VC155 alone (data not shown). Fluorescence was observed when cells were co-transfected with the p62–VN173 and VHL–VC155 vectors (Fig. S3A), suggesting that p62 and VHL heterodimers form in cells. We further confirmed the interaction between endogenous p62 and the VHL E3 ubiquitin ligase complex. Immunoprecipitation of endogenous p62 from HEK293T cells resulted in the co-precipitation of VHL and CUL2 but not of HIF1α (Fig. 4C), further supporting the notion that this complex is present in cells.

To identify the domain of VHL that interacts with p62, we generated Flag-fused recombinant full-length and truncated mutants of human VHL (Fig. 4D, left). Domain-mapping experiments indicated that the intact β-domain of VHL had an ability to associate with p62 (Fig. 4E). As shown in Fig. 4E, p62 possesses seven domains (Komatsu et al., 2010). To determine which domain of p62 is required for its interaction with CUL2 and VHL, we prepared a deletion series of p62 constructs (Fig. 4E, right) and employed a co-immunoprecipitation assay using HA–VHL and Myc–CUL2. The HA–VHL and Myc–CUL2 proteins were clearly detected in precipitates of both full-length p62 and the p62 mutants harboring the TRAF6-binding domain (residues 170–260) (Fig. 4F, lanes 1–3 and 5). However, p62 deletion mutants that lacked the TRAF6-binding domain showed a marked decrease in binding to HA–VHL and Myc–CUL2 (Fig. 4F, lanes 4 and 6–8). These results therefore suggest that residues 170–260 of p62 are essential for its molecular recognition of VHL and CUL2, as is the case for the recognition of TRAF6.

**Fig. 5.**
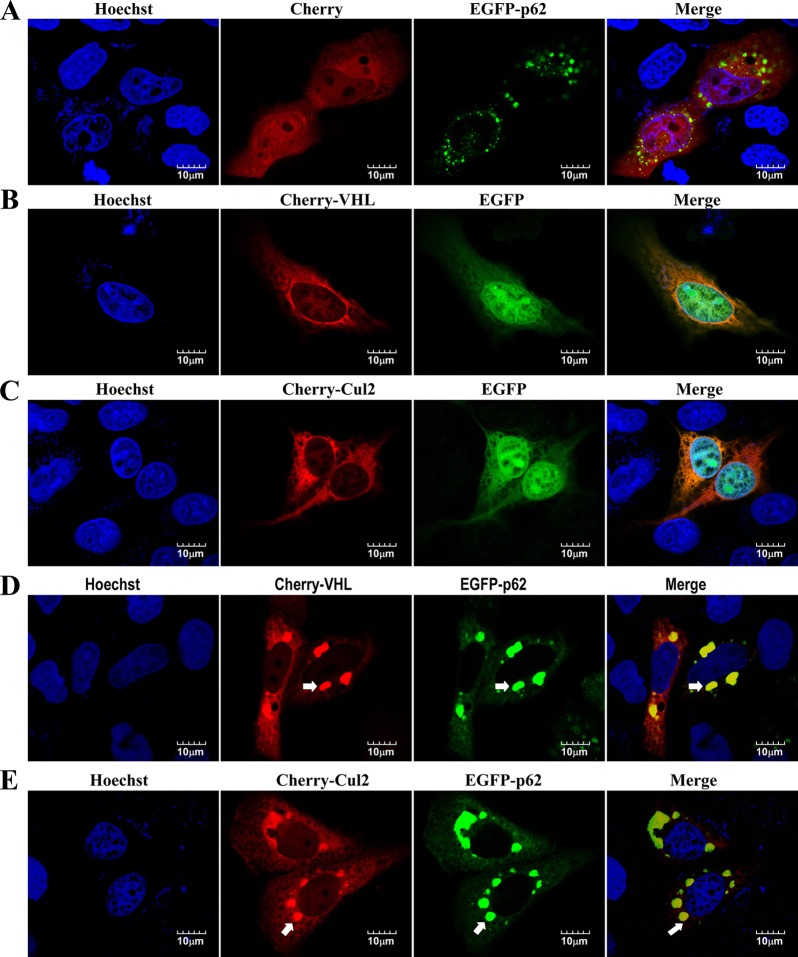
**Colocalization of p62 with CUL2 and VHL.** (A–E) Caki-1 cells were transfected with different combinations of expression vectors with pmCherry-C1 (control), mCherry–VHL, mCherry–CUL2, pEGFP-C1 and EGFP–p62. After transfection for 48 h, the cells were treated with Hoechst 33258 to stain the nucleus and observed by using confocal microscopy. Arrows indicate colocalization in aggregates. Scale bars: 10 µm.

**Fig. 6.**
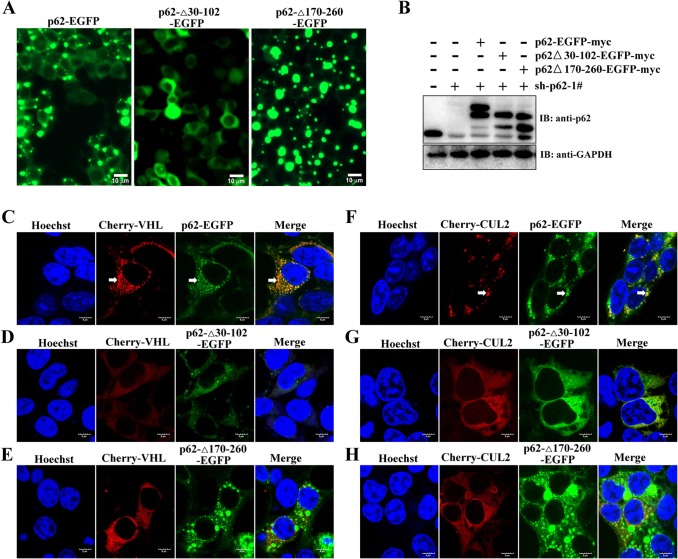
**Interaction with p62 is required for VHL and p62 aggregation.** (A,B) Generation of p62-knockdown cells that were engineered to re-express p62–EGFP, p62Δ30-102–EGFP or p62Δ170-260–EGFP. HEK293T cells were infected with lentivirus expressing shRNA against p62 (sh-p62-1#), and then p62–EGFP, p62Δ30-102–EGFP or p62Δ170-260–EGFP was expressed in the knockdown cells to establish stably expressing cell lines. (A) Images were taken with a fluorescence microscope. Scale bars: 10 µm. (B) Immunoblotting of lysates derived from these engineered cells, using antibodies against p62 and GAPDH. (C–H) The engineered cell lines were infected with lentivirus encoding mCherry–VHL or (F–H) mCherry–CUL2. After transfection for 48 h, the cells were stained with Hoechst 33258 and observed by using confocal microscopy. Arrows indicate colocalization in aggregates. Scale bars: 5 µm.

p62 is found in cytosolic aggregates, which contain a number of proteins, including polyubiquitylated proteins (such as aPKC and VDAC1), PHD3 and components of the proteasomal system (Rantanen et al., 2013). Consistent with previous reports (Katsuragi et al., 2015), subcellular localization and western blot analysis indicated that most p62 protein was distributed in the cytoplasm, but it also localized in the nucleus (Fig. S3B,C). Consistent with the results of the BiFC analysis (Fig. S3A), overexpressed mCherry–VHL and p62–EGFP colocalized in the aggregates in Caki-1 cells, whereas mCherry alone failed to colocalize with p62 aggregates (Fig. 5A and D). Similar to mCherry–VHL, mCherry–CUL2 colocalized with p62–EGFP in aggregates (Fig. 5E). To rule out that the aggregation of VHL and CUL2 was due to p62–EGFP overexpression, we used p62-knockdown cells and then re-introduced p62–EGFP, p62Δ30-102–EGFP (deletion of the PB1 domain) or p62Δ170-260–EGFP into the knockdown cells to restore p62 expression to wild-type levels (Fig. 6A,B). Next, these engineered cells were transfected with mCherry–VHL or mCherry–CUL2 by using lentiviral vector. Cells expressing mCherry–VHL and mCherry–CUL2 showed the expected punctuate expression pattern, which colocalised with that of p62–EGFP (Fig. 6C,F). As expected, aggregates almost completely failed to form in cells expressing p62Δ30-102–EGFP (Fig. 6A). Importantly, mCherry–VHL and mCherry–CUL2 showed a diffuse expression pattern in p62Δ30-102–EGFP-expressing cells (Fig. 6D,G). Interestingly, p62Δ170-260–EGFP is a mutant that disrupts the interaction between VHL and CUL2, and this mutant results in the formation of larger aggregates than those formed in p62–EGFP-expressing cells. However, mCherry–VHL and mCherry–CUL2 failed to form aggregates in p62Δ170-260–EGFP-expressing cells (Fig. 6E,H). Taken together, these data demonstrate that the interaction with p62 is needed for the aggregation of VHL and CUL2.

### p62 blocks the VHL-mediated ubiquitylation of HIFα

**Fig. 7.**
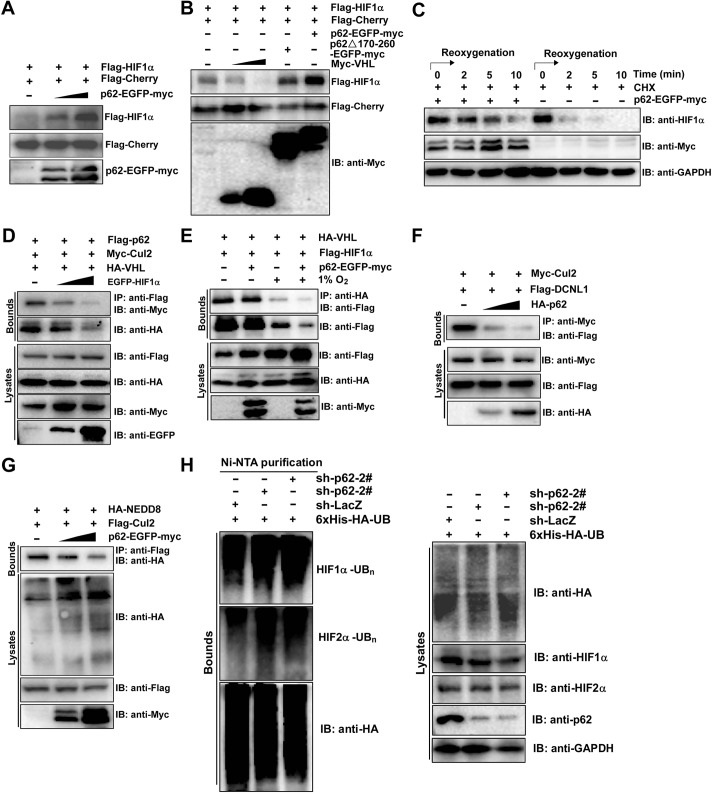
**p62 stabilizes HIF1α by negatively regulating VHL ubiquitin ligase activity.** (A) p62 increases the HIF1α protein levels. HEK293T cells were co-transfected with Flag–mCherry (0.2 μg), Flag–HIF1α (0.2 μg) and p62–EGFP–Myc (0, 0.2 or 0.6 μg) plasmids. Cell lysates were subjected to SDS-PAGE followed by immunoblotting with anti-Flag and anti-Myc antibodies. (B) The VHL- and CUL2-binding region of p62 is necessary for p62-mediated increases of HIF1α protein levels. HEK293T cells were co-transfected with Flag–mCherry (0.2 μg), Flag–HIF1α (0.2 μg) together with Myc–VHL (0, 0.2 or 0.6 μg), p62Δ170-260–EGFP–Myc (0.6 μg) or p62–EGFP–Myc (0.6 μg). Cell lysates were subjected to SDS-PAGE followed by immunoblotting with anti-Flag and anti-Myc antibodies. (C) p62 increases the stability of the endogenous HIF1α protein. ACHN cells expressing p62–EGFP–Myc or parental ACHN cells were incubated under hypoxia (12 h) and then moved to normoxia (time 0) with the addition of cycloheximide (CHX, 50 μg/ml). Cell lysates were subsequently harvested at sequential time points (0, 2, 5 or 10 min) after treatment, and then the cell lysates were immunoblotted for HIF1α, Myc or GAPDH. (D) p62 binds to the VHL complex and competes with HIF1α. HEK293T cells were co-transfected with Flag–p62, Myc–CUL2, HA–VHL and EGFP–HIF1α (0, 0.4 or 1 μg) plasmids. Cell lysates were precipitated (IP) with anti-Flag antibody and immunoblotted (IB) for Myc and HA. (E) p62 attenuates the interaction between VHL and HIF1α. HEK293T cells were co-transfected with HA–VHL, Flag–HIF1α and/or p62–EGFP–Myc plasmids. At 24 h post transfection, cells were maintained under normoxia or exposed to hypoxia for 12 h. Cell lysates were precipitated with an anti-HA antibody and immunoblotted for Flag. (F) p62 competes with DCNL1 for binding to CUL2. HEK293T cells were transfected with Myc–CUL2 and Flag–DCNL1 together with an increasing amount of HA–p62 (0, 0.4 or 1 μg). Cell lysates were precipitated with anti-Flag antibody and immunoblotted for Myc. (G) p62 attenuates NEDD8-mediated modification of CUL2. HEK293T cells were co-transfected with Myc–CUL2 and HA–NEDD8, and an increasing amount of Flag–p62. Cell lysates were immunoprecipitated with an anti-Myc antibody and immunoblotted with the indicated antibodies. (H) p62 inhibits endogenous ubiquitylation of both HIF1α and HIF2α. HEK293T cells were infected with lentivirus expressing His–HA–ubiquitin (6×His-HA-UB). After 72 h in culture, cells were treated with 10 µM MG-132 for 12 h, and subsequently protein lysates were prepared in urea buffer. Ni-NTA affinity purifications were used to pull down only proteins covalently modified with 6×His-HA-tagged ubiquitin. Western blots of protein lysates and purified Ni-NTA agarose elutions were immunoblotted for HA (ubiquitin), HIF1α, HIF2α and p62.

To examine the effect of p62 on the stability of HIF1α, we transfected HEK293T cells with Flag–mCherry and Flag–HIF1α, along with increasing amounts of p62–EGFP–Myc. After normalization of the expression of Flag–mCherry in cell lysates, we found that the protein levels of Flag–HIF1α increased in p62-transfected cells in a dose-dependent manner (Fig. 7A). Moreover, the protein level of co-expressed Flag–HIF1α was not obviously increased in p62Δ170-260-transfected cells (Fig. 7B), suggesting that the VHL- and CUL2-binding region of p62 plays an important role in enhancing the HIF1α protein levels. Because p62 has an important role in transporting polyubiquitylated proteins to the autophagosomes and proteasome for degradation (Rantanen et al., 2013), we then examined the mechanism by which p62 regulates the protein stability of HIFα. Unlike the proteasome inhibitor MG-132, lysosomal autophagy inhibitors chloroquine or baﬁlomycin A1 were unable to elevate both HIF1α and HIF2α levels (Fig. S4A), indicating that p62 increases HIF protein levels mainly by inhibiting ubiquitin-dependent proteolysis of the HIF proteins. Furthermore, overexpression of p62 reduced the rate of HIF1α degradation in p62-knockdown ACHN cells in experiments using cycloheximide (Fig. 7C). It is known that hydroxylated HIF1α is recognized by the β-domain of VHL. Thus, we speculate that p62 competes with HIF1α for binding to the VHL complex. As expected, when HIF1α was transiently overexpressed, it interfered with the CUL2–p62 and VHL–p62 interactions in a dose-dependent manner (Fig. 7D). Furthermore, the interaction between HIF1α and VHL was also decreased in HEK293T cells that overexpressed p62, both under normoxia and hypoxia (Fig. 7E), indicating that less HIF1α is recruited to VHL in the presence of p62. Because conjugation of Nedd8 to CUL2 (neddylation) is crucial for activation of CUL2, and the engagement of the substrate HIF1α with VHL prompts DCNL1 recruitment and then initiates CUL2 neddylation (Heir et al., 2013), we studied whether p62 regulates CUL2 neddylation. Co-immunoprecipitation assays showed that p62 inhibited the DCNL1–CUL2 interaction in a dose-dependent manner (Fig. 7F). Furthermore, p62 inhibited the neddylation of CUL2 in a dose-dependent manner (Fig. 7G). These results indicate that p62 inhibits both the HIF1α–VHL interaction and CUL2 neddylation by binding to VHL E3 ubiquitin ligase. We further examined whether p62 affects HIFα ubiquitylation. As expected, decreased levels of ubiquitylated HIF1α and HIF2α were observed in p62-overexpressing HEK293T cells (Fig. S4B,C). To demonstrate that endogenous ubiquitylation of HIF1α and HIF2α is regulated by p62, we generated HEK293T cells that stably expressed 6×His-tagged wild-type ubiquitin through lentiviral transfer of 6×His–HA–ubiquitin plasmids. Indeed, the levels of HIF1α and HIF2α high-molecular-mass species were both increased upon p62 knockdown under normoxia (Fig. 7H). Taken together, these results suggest that p62 competes with HIF1α in binding to the VHL E3 ubiquitin ligase complex and inhibits neddylation of CUL2, thereby inhibiting ubiquitylation of HIFα.

Collectively, these experiments establish that p62 increases HIF1α levels by both upregulating HIF1α mRNA and inhibiting degradation of the protein. To assess the contribution of the two mechanisms to p62-mediated HIF1α accumulation, we evaluated the mRNA, protein and transcriptional activities of HIF1α under both normoxia (20% O_2_) and hypoxia (5% O_2_ or 1% O_2_). Under normoxic conditions, we observed a significant decrease in the mRNA levels of HIF1α in p62-knockdown cells (48% of HIF1α mRNA as compared to that of control cells). At 1% O_2_, knockdown of p62 only resulted in a 16% decrease in HIF1α mRNA relative to that of control cells (Fig. S4D). Furthermore, the HIF1α protein levels were also decreased in p62-knockdown cells under hypoxic conditions (Fig. S4E). However, forced p62 expression significantly increased HIF1α expression (about 50% as compared with control cells) under both normoxic and hypoxic conditions (Fig. S4F). A possible explanation for the inconsistent effect of p62 knockdown and overexpression on the HIF1α levels under hypoxia is that the low expression of p62 protein as a result of hypoxia-activated autophagy accelerates its degradation. Indeed, the protein levels of p62 were obviously downregulated under hypoxia (Fig. S4E,G). In addition, re-introducing p62 into stable p62-knockdown cells induced transcriptional activities of HIF in both normoxic and hypoxic cells (Fig. S4H). Because NF-κB plays an important role in mediating p62-induced HIF1α transcription, we constructed double p65- and p62-knockdown cells. Next, the engineered cells were cultured under hypoxia or normoxia for 12 h, either with or without expression of Flag–p62. As expected, Flag–p62 expression had little effect on the mRNA levels of HIF1α in both normoxia and hypoxia, whereas the protein levels of HIF1α were obviously upregulated under normoxia (Fig. S4I,J; Fig. 3D, lanes 2 and 4). Furthermore, forced expression of p62 in double p62- and p65-knockdown cells also increased HRE-luciferase reporter activity under both normoxia and hypoxia (Fig. S4K), suggesting that the effect of p62 on HIF1α accumulation is mediated, at least in part, through the enhancement of HIF1α stability under normoxic conditions. More importantly, the increase in the transcriptional activity of HIF1 was noticeable upon exogenous expression of p62, and the effect was less prominent in (p62 and p65) double-knockdown cells than in p62 single-knockdown cells, especially under hypoxic culture conditions, meaning that the p62-induced upregulation of HIF1α expression might be a major contributor to HIF1α accumulation under hypoxic conditions (Fig. S4K). Taken together, these results support the concept that both p62-mediated upregulation of HIF1α mRNA and increased HIF1α protein stability are required for maximum induction of HIF1α activity.

### HIF1α contributes to the function of p62 in regulating the Warburg effect and growth in soft agar

**Fig. 8.**
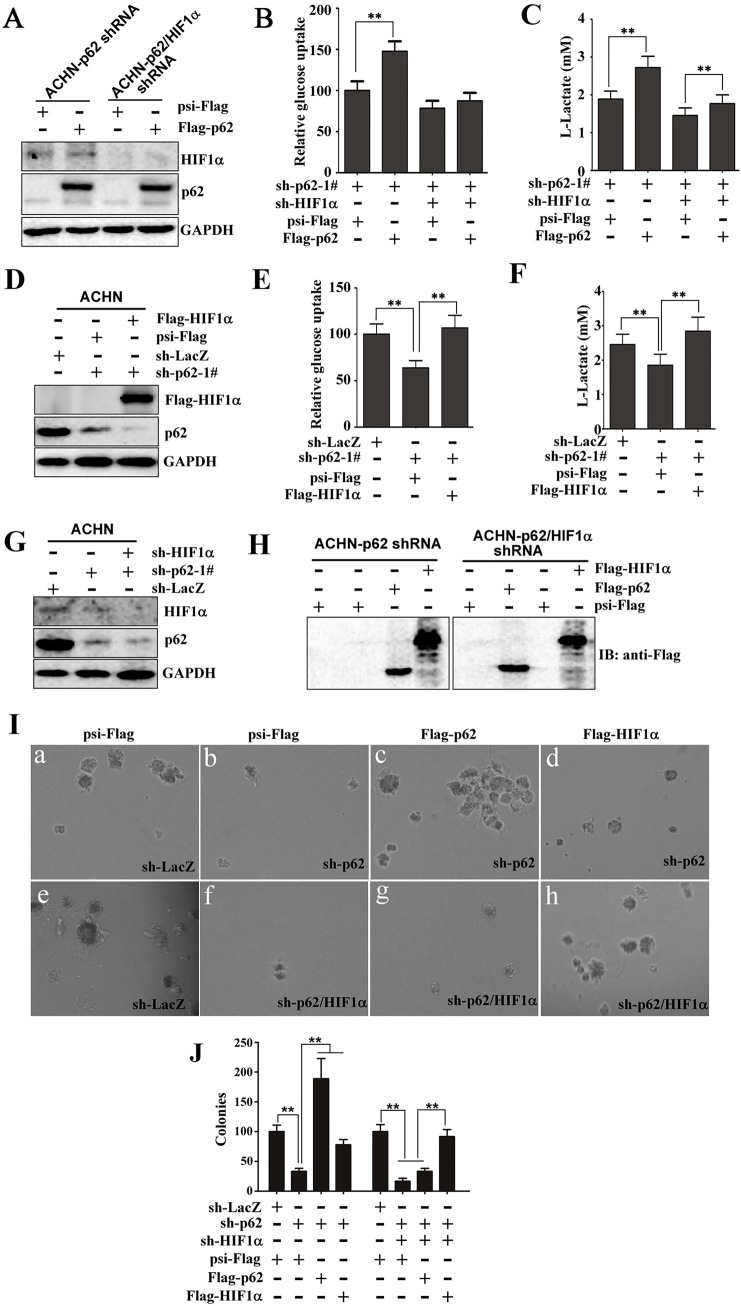
**HIF1α contributes to the function of p62 in regulating the Warburg effect and growth in soft agar.** (A) Total protein was extracted from ACHN cells that stably expressed sh-p62 and/or sh-HIF1α, followed by western blotting to evaluate the expression of HIF1α. GAPDH is an internal control. (B,C) Knockdown of HIF1α severely impairs p62-mediated increased glucose uptake and lactate production. ACHN cells that stably expressed sh-p62-1# and/or sh-HIF1α were infected with lentivirus expressing Flag–p62. After transfection for 72 h, glucose uptake (B) and extracellular lactate in the culture medium (C) were measured and expressed as a ratio of those in sh-LacZ cells. Significant differences, **P<0.01 (Student's *t*-test); mean±s.d. *n*=3. (D) Immunoblotting for HIF1α, Flag and GAPDH (loading control) of lysates derived from ACHN cells that stably expressed sh-p62 and/or sh-HIF1α and that had then been infected with lentivirus expressing Flag–HIF1α. (E,F) The effect of silencing p62 on glucose uptake and lactate production was reversed by HIF1α overexpression. Cells were treated as described in D. (E) Glucose uptake and (F) extracellular lactate in the culture medium were measured. **P<0.01 Student's *t*-test); mean±s.d. *n*=3. (G,H) Generation of ACHN cells that stably expressed shRNA against p62 (sh-p62-1#) or against HIF1α (sh-HIF1α) (G), which were then infected with lentivirus encoding psi-Flag (Control), Flag–p62 or Flag–HIF1α (H). Western blotting analysis of the lysates from these engineered cells was performed to evaluate the expression of HIF1α, p62, Flag–p62 and Flag–HIF1α. GAPDH is an internal control. (I) p62 regulates colony growth in soft agar through HIF1α. ACHN cells in which p62 or p62 and HIF1α had been knocked down were infected with lentivirus expressing psi-Flag (control), Flag–p62 or Flag–HIF1α. After transfection for 48 h, the cells were plated in soft agar. The number of colonies was measured after 14 days. (Ia–Ih) Representative photographs were taken by using an inverted microscope. (J) The number of colonies was quantified in three random images from each treatment group. Results are the mean±s.d. from three independent experiments, plotted as the percentage of colonies formed by sh-LacZ-expressing cells. Significant differences, **P<0.01 (Student's *t*-test).

Multiple studies have shown that some HIF1 target genes regulate processes involved in glucose uptake and glycolysis, and oncogenic transformation (Kaelin, 2008; Semenza, 2010). One of the most important questions we wanted to investigate is whether or not HIF1α contributes to p62-induced aerobic glycolysis. To answer this question, we performed shRNA depletion and overexpression experiments to assess the effect on p62-mediated increased glucose uptake and lactate production. As expected, HIF1α protein levels were reduced in cells that stably expressed sh-HIF1α (Fig. 8A, lanes 3 and 4). Re-introduction of p62 into the p62-knockdown cells increased glucose uptake and lactate production (Fig. 8B,C). However, the presence of sh-HIF1α severely impaired p62-increased glucose uptake and lactate production (Fig. 8B,C). Additionally, we also examined whether HIF1α overexpression affects the inhibitory of the Warburg effect by p62 knockdown (Fig. 8D). We found that the effect of p62 silencing on glucose uptake (Fig. 8E) and lactate production (Fig. 8F) was reversed by overexpressing HIF1α. Taken together, these findings indicate that expression of HIF1α is crucial for p62-enhanced glucose uptake and lactate production.

It has been reported that p62 promotes colony formation in soft agar by regulating the mTORC1 and NF-κB pathways (Duran et al., 2008; Li et al., 2013b). We next sought to determine whether HIF1α mediates p62-induced growth in soft agar. We evaluated this possibility by constructing a series of ACHN cell lines through lentiviral infection with different combinations of sh-p62, sh-HIF1α, Flag–p62, and Flag–HIF1α. As expected, p62 and HIF1α protein levels were reduced in cells that stably expressed the knockdown constructs sh-p62 and sh-HIF1α, respectively (Fig. 8G). The overexpression of Flag–p62 or Flag–HIF1α in cells was also verified by western blotting (Fig. 8H). We found that p62 knockdown decreased colony formation by ACHN cells in soft agar (Fig. 8Ib), consistent with a previous report (Li et al., 2013b). Consistently, p62 re-expression in ACHN cells that expressed sh-p62 restored colony growth (Fig. 8Ic). Interestingly, ectopic HIF1α expression in ACHN cells that expressed sh-p62 partially restored the ability to form colonies (Fig. 8Id). These results suggest that the promotion of growth in soft agar by p62 involves at least one effector other than HIF1α. Furthermore, simultaneous knockdown of p62 and HIF1α resulted in an almost complete loss of colony growth in soft agar (Fig. 8If). Moreover, the inhibitory effect of double p62- and HIF1α-knockdown on colony formation in soft agar was largely reversed by HIF1α overexpression but not by p62 overexpression (Fig. 8Ie–Ih), meaning that HIF1α acts downstream to p62. Quantification of colonies derived from the genetically modified ACHN cells is shown in Fig. 8J. Collectively, these data show that HIF1α expression is important for p62-induced colony growth in soft agar.

## DISCUSSION

In the present study, we have explored a previously uncharacterized relationship between p62 and HIF1α, and demonstrated that p62 regulates energy metabolism and colony formation in soft agar by regulating HIF1α levels in renal cancer cells. Our data show that p62 positively affects mTORC1 activity and the nuclear translocation of NF-κB (Rius et al., 2008; Duran et al., 2011; Linares et al., 2013), leading to increased HIF1α levels. Notably, here we show that p62 binds to the VHL E3 ligase complex, blocks activity of the E3 ligase and thus increases HIF1α protein stabilization. This evidence strongly suggests that p62 regulates the tumor-promoting ability of HIF1α.

It is known that HIF1α plays a key role in the reprogramming of metabolism in cancer. High HIF1α expression is associated with mortality from cancers of the bladder, brain, breast, colon, cervix, endometrium, head and neck, lung, ovary, pancreas, prostrate, rectum and stomach (Young and Simon, 2012). Many studies strongly support that HIF1α functions as a tumor-promoting gene (Semenza, 2012). Loss of chromosome 3p, loss of chromosome 14q (harbors HIF1α) and gain of chromosome 5q (harbors p62) are the three most common genomic abnormalities in kidney cancer (Shen et al., 2011). A considerable amount of evidence shows that HIF1α plays a tumor-suppressive role in VHL-defective renal carcinogenesis (Shen et al., 2011; Young and Simon, 2012). However, HIF2α shows a key role in promoting VHL-deficient renal cancers (Keith et al., 2012). At present, the molecular mechanisms underlying this ambiguity are not well understood. The opposing effects of HIF1α and HIF2α in renal cancer could be the result of their different effects on Myc activity (Young and Simon, 2012). By contrast, Myc has a role in the induction of HIF1α protein levels, and cooperates with HIF1 to induce glycolysis and tumor growth (Doe et al., 2012). Thus, the pathways of HIF1α and of Myc are interlinked, and both genes regulate tumor promotion and progression. p62 is also involved in regulating Myc activity though mTORC1 (Valencia et al., 2014), and Myc can stabilize HIFα (Doe et al., 2012); thus the pathways of p62–VHL–HIFα and p62–mTORC1–Myc–HIFα might be parallel, or intertwined. Here, we demonstrate that p62 upregulates HIF1α mRNA and protein levels by regulating the activity of mTORC1, NF-κB and the VHL complex. Previous studies have shown mTORC1 and NF-κB play key roles in promoting translation and transcription of HIF1α, respectively (Thomas et al., 2006; Rius et al., 2008; van Uden et al., 2008). In addition, an increasing number of studies has demonstrated that p62 expression upregulates mTORC1 activity (Duran et al., 2008; Linares et al., 2013; Valencia et al., 2014). p62 also plays key role in the activation of NF-κB in several cell systems (Sanz et al., 1999, 2000; Wooten et al., 2005; Duran et al., 2008; Nakamura et al., 2010); however, the many possible pathways in which p62 and HIF1α could interact remain to be clarified. For example, previous results have shown that p62 represses the generation of reactive oxygen species (ROS) by inducing the activity of NF-κB, NRF2 and/or the mTORC1–Myc pathway (Duran et al., 2008; Li et al., 2013b; Valencia et al., 2014). Meanwhile, elevated levels of ROS or Myc have been shown to promote increased HIF1α activity (Faubert et al., 2014). Thus, it is possible that p62 regulates HIF1α through NRF2–ROS, NF-κB–ROS and/or mTORC1–Myc–ROS cascades.

Importantly, we demonstrated that p62 induces a dramatic enhancement in the stability of both the HIF1α and HIF2α proteins. A previous study has reported that p62 interacts with CUL2, but the molecular mechanism of this interaction has not been studied (Tanji et al., 2012). It is well known that CUL2 neddylation is important for its E3 ligase activity. DCNL1 mediates CUL2 neddylation upon engagement with HIF1α (Heir et al., 2013). We provide the first evidence that p62 binds to and inhibits the activity of VHL ubiquitin ligase by inhibiting CUL2 neddylation. Furthermore, p62 binds to VHL and competes with HIFα for VHL binding. Therefore, the upregulation of p62 in some types of tumor probably results in dissociation of HIFα from the VHL E3 ligase complex and the formation of a heterodimer with HIFβ, followed by transcriptional activation of HIF target genes. It has been established that p62 interacts with Keap1, a component of Cullin-3 (CUL3) ubiquitin ligase complexes, and p62 is also a substrate of CUL3 (Komatsu et al., 2010; Lee et al., 2012). In addition, p62 competes with NRF2 for Keap1 binding, resulting in stabilization of NRF2 (Komatsu et al., 2010; Inami et al., 2011). It is interesting to note that p62 can function in both CUL2- and CUL3-type ubiquitin ligase pathways. As NRF2 enhances HIFα stability by promoting mitochondrial O_2_ consumption (Kim et al., 2011), it will be interesting to test whether CUL3 and/or NRF2 also cooperates with p62 in the regulation of HIF1α.

Our study is the first demonstration that the oncogenic properties of p62 are due to its effects on the HIF pathway. We demonstrated that p62 is a potent activator of the Warburg effect, promoting the production of lactic acid and glucose uptake. p62 also markedly promoted colony growth of renal cancer cells in soft agar. Yet multiple lines of evidence suggest that HIF1α functions as a tumor suppressor, which is inactivated in a high percentage of ccRCCs (Shen et al., 2011); in contrast, HIF1α is believed to promote the tumorigenic properties of many other tumors, making it difficult to understand how HIF1α might differentially function in tumorigenesis of different cell types. A possible explanation for the inconsistent pro- or anti-tumorigenic effects of HIF1α in some tumor types is that its function is highly dependent on the specific cellular context. In addition, inappropriate levels of HIF activity might be an important determinant in tumor growth. For example, both overexpression and inhibition of HIF2α can promote growth of non-small-cell lung cancer (Mazumdar et al., 2010). It is also interesting that p62 can exert tumor-suppressive functions and has been shown to be downregulated in the stroma of several tumors (Valencia et al., 2014). These suggest that both p62 and HIF1α exert either pro- or anti-tumorigenic effects in a cell-type- and -context-dependent manner; further investigation is required to assess the effect of p62-related diverse cascades, such as p62–mTORC1–Myc, p62–NFκB–HIF1α and p62–VHL–HIF2α–Myc, on the progression of cancer.

In summary, we have described several pathways that link p62 to HIF1α, which could be potentially involved in many molecular processes. Identification of the relationship between p62, CUL2 and HIF will be undoubtedly helpful for our understanding of the molecular mechanisms underlying tumorigenesis.

## MATERIALS AND METHODS

### Antibodies

The following antibodies were used in the experiments: anti-Flag (F3165, 1:2000) from Sigma-Aldrich; anti-GFP (11814460001, 1:2000), anti-Myc (11667149001, 1:2000) and anti-HA (11583816001, 1:2000) from Roche Applied Science; anti-CUL2 (ab166917, 1:500) and anti-GLUT1 (ab115730, 1:1000) antibodies from Abcam. Anti-p62 (#8025, 1:1000), anti-HIF1α (#3716, 1:200), anti-p65 (#6956, 1:1000), anti-p70S6K (#9202, 1:1000), anti-phospho-p70S6K (Thr389) (#9205, 1:1000), anti-VHL (#2738, 1:500) antibodies were from Cell Signaling Technology; anti-HIF1α (NB100-105, 1:500) and anti-HIF2α (NB100-122, 1:500) antibodies were from Novus Biologicals (Littleton, CO); anti-VHL (clone D-7) antibody was from Santa Cruz Biotechnology; anti-GAPDH (CW0100, 1:2000) antibody was purchased from Beijing CWBio (CWBIO, Peking, China); goat anti-­mouse IgG horseradish peroxidase (HRP)-­conjugated whole antibody (31430, 1:50,000) and goat anti-­rabbit IgG-HRP-­conjugated whole antibody (31460, 1:50,000) was purchased from Thermo Scientific. Cy3-labeled goat anti-rabbit or mouse IgG was purchased from CWBio.

### Plasmid constructs

The full-length and deletion mutants of VHL have been described previously (Chen et al., 2015). Flag–elonginB, Flag–elonginC, Myc–CUL2, Flag–CUL2, mCherry–CUL2, HA–ubiquitin, Flag–HIF1α and EGFP–Myc–HIF1α plasmids were from Prof. Rongjia Zhou (Wuhan University, China). Mammalian expression plasmids for human HA-, Flag- or GFP-, mCherry-, Venus-N173-tagged p62 were constructed using standard molecular biology techniques with primers p62-5′ and p62-3′ (all primers are detailed in Table S1). To obtain the p62 fragment comprising residues 1–117, 1–266, 260–345 and 345–440, HA–p62 was PCR amplified using primers p62-5′ and p62-N1-3′, p62-5′ and p62-N2-3′, p62-M-5′ and p62-M-3′, p62-C-5′ and p62-3′, respectively. The p62 deletion mutants Flag–p62Δ30-102, Flag–p62Δ128-163 and Flag–p62Δ170-260 were generated by using a two-step PCR-based mutagenesis procedure using HA–p62 as the template. First-step PCR was used to amplify two partially overlapping fragments using primers p62-5′ plus p62-dPB1-3′ and p62-dPB1-5′ plus p62-3′, p62-5′ plus p62-dZZ1-3′ and p62-dZZ1-5′ plus p62-3′, p62-5′ plus p62-dT1-3′ and p62-dT-5′ plus p62-3′. The fragments were annealed and used as the template for second-step PCR with the primer p62-C-5′ and p62 -3′ to obtain the p62Δ30-102, p62Δ128-163 and p62Δ170-260 mutants, respectively. p62–EGFP–Myc, p62Δ30-102–EGFP–Myc and p62Δ170-260–EGFP–Myc recombinants were constructed by subcloning the coding region of full-length p62 and mutants into *Xho*I and *Bam*HI sites of psi-EGFP-myc-N1. p62–VN173 was constructed by cloning the coding region of p62 into *Xho*I and *Bam*HI sites of pVN173-N3 (a gift from Dr Xiaodong Zhang, Wuhan University). VHL-VC155 was constructed by cloning the coding region of VHL into *Xho*I and *Bam*HI sites of pVC155-N3. Human cDNA was PCR amplified using primers NEDD8-5′ and NEDD8-3′, digested by *Eco*RI and ligated into psi­HA­C1 to create HA–NEDD8. Human cDNA was PCR amplified using primers DCNL1-5′ and DCNL-3′, digested by *Bam*HI and *Xho*I, and ligated into psi-Flag-C1 to create Flag–DCNL1. Human cDNA was PCR amplified using primers HIF2A-5′ and HIF2A-3′, digested by *Bam*HI and *Sal*I, and ligated into psi-Flag-C1 to create Flag–HIF2α. 6×His–HA–ubiquitin was constructed by subcloning the encoding the region of HA–ubiquitin into the *Nhe*I and *Eco*RI sites of pSicoR-GFP (Addgene). HA–ubiquitin was PCR amplified using HIS-5′ and UB-3′, digested by *Xba*I and *Mun*I, and ligated into pSicoR-GFP. Lentiviral 6×HRE-Fluc and lentiviral SV40-Rluc, which contain a lentiviral vector backbone, were constructed by subcloning the 6×HRE mini-promoter and the region encoding firefly luciferase of pGL4-6×HRE-Luc2 (Qiagen) and SV40 promoter and *Renilla* luciferase CDS into the *Xba*I and *Xho*I sites of psi-Flag-C1, respectively. pGL4-6×HRE-Luc2 and pRL-SV40 (Promega) were PCR amplified using primers pGL4-5′ plus Luc2-3′ and SV40-5′ plus Rluc-3′, respectively. The products were digested with *Xba*I plus *Sal*I or *Xho*I and ligated into psi-Flag-C1. PCR conditions and primers for the creation of these constructs are provided in Table S1. All constructs were verified by sequencing.

### RNA interference

Oligonucleotides corresponding to the target sequences were annealed and cloned into the *Hpa*I and *Xho*I sites of the pSicoR-Puro plasmid. The following target regions were chosen: p62-1#, 5ʹ-GGGCATCCGCAATGTTGGT-3ʹ; p62-2#, 5ʹ-GGACCCATCTGTCTTCAAA-3ʹ; HIF1α, 5ʹ-GTCTGCAACATGGAAGGTA-3ʹ; p65, 5ʹ-GTGCCTTAATAGTAGGGTAAG-3ʹ. PCR conditions and primers for the creation of these constructs are provided in Table S1. All constructs were verified by sequencing.

### Cell culture

ACHN, Caki-1 and HEK293T cells were cultured in Dulbecco's modified Eagle's medium (DMEM; Invitrogen Life Technologies) with 10% fetal bovine serum (HyClone) under 5% CO_2_ at 37°C in a humidified incubator (Thermo Fisher Scientific). For hypoxic treatment, cell culture dishes were placed into a New Brunswick Galaxy^®^ CO_2_ incubator (New Brunswick Scientific) at the indicated oxygen concentrations. Autophagy inhibitors 3-methyladenine (3-MA, Sigma-Aldrich) and bafilomycin A1 (Sigma-Aldrich), and proteasome inhibitor MG-132 (Sigma-Aldrich) were used at the indicated concentrations.

### Isolation of ubiquitylated proteins using Ni-NTA superflow resin

HEK293T cells stably expressing 6×His-HA-UB were lysed in lysis buffer (10 mM Tris-HCl at pH 8.0, 100 mM NaH_2_PO_4_ and 8 M urea) containing 10 mM imidazole. After centrifugation, supernatant was incubated with Ni-NTA Superflow agarose (Pierce Biotechnology) for 4 h at RT. The resins were washed four times with lysis buffer containing 20 mM imidazole. After elution with loading buffer (3% SDS, 1.5% mercaptoethanol, 8% glycerol, 0.01% Coomassie Blue G-250, 150 mM Tris–HCl pH 7.0) at 50°C for 30 min, the bound proteins were then subjected to western blot analysis.

### Glucose uptake and lactate production

Glucose uptake was examined using a glucose uptake cell-based assay kit (item number 600470, Cayman Chemical, MI). ACHN and Caki­1 cells were infected with lentivirus for 72 h, and then cells were digested and transferred to 96-well black clear-bottom culture plates with 3×10^4^ cells/well in 100 μl of culture medium. Approximately 24 h later, the glucose uptake was measured by using the glucose uptake cell-based assay kit. Lactate production was determined with a glycolysis assay kit (item number 601060, Cayman Chemical). ACHN and Caki-1 cells were transferred to a 96-well plate at a density of 3×10^4^ cells/well in 200 μl of culture medium. The lactate production was determined using the glycolysis assay kit according to manufacturer's instructions.

### Soft agar colony formation assay

ACHN cells were infected with lentivirus for 48 h, then cells were seeded into 0.35% agar Noble (Sigma-Aldrich) in DMEM containing 10% FBS on top of a bed of 0.5% agar in a 6-well plate at a density of 2000 cells/well. Agar at 42°C was mixed with medium at 37°C, plated and left to set for 10 min. After 14 days, the number of colonies was scored using an inverted microscope.

### Other methods

Transfection, virus generation and infection, RT-qPCR, luciferase activity assays, co-immunoprecipitation assays, ubiquitylation and cycloheximide chase assays, isolation of cytosolic and nuclear proteins and cell proliferation assays were performed as described previously (Chen et al., 2013, 2015).

### Statistical analysis

The data are presented as the means±s.d. Comparisons between two groups were performed using an unpaired Student's *t*-test. A value of *P*<0.05 was considered significant.
